# Editorial: Recent Advances in Application of Synthetic Biology for Production of Bioactive Compounds

**DOI:** 10.3389/fbioe.2021.819475

**Published:** 2021-12-22

**Authors:** Luan Luong Chu, Jingwen Zhou, Dipesh Dhakal, Jae Kyung Sohng

**Affiliations:** ^1^ Faculty of Biotechnology, Chemistry and Environmental Engineering, Phenikaa University, Hanoi, Vietnam; ^2^ Bioresource Research Center, Phenikaa University, Hanoi, Vietnam; ^3^ Science Center for Future Foods, Jiangnan University, Wuxi, China; ^4^ Department of Medicinal Chemistry, University of Florida, Gainesville, FL, United States; ^5^ Department of Life Science and Biochemical Engineering, SunMoon University, Chungnam, South Korea; ^6^ Department of Pharmaceutical Engineering and Biotechnology, SunMoon University, Chungnam, South Korea

**Keywords:** synthetic biology, metabolic engineering, bioactive compounds, *E. coli*, *S. cerevisiae*

Bioactive natural compounds broadly exhibit application in various fields, as chemical and food additives, agrochemical products, cosmetics, pharmaceuticals, and biofuels. Furthermore, unnatural bioactive compounds have been synthesized and shown a great effect on promoting better health. Both natural and unnatural bioactive compounds include polyphenols, flavonoids, terpenoids, alkaloids, carotenoids, stilbenes, and anthocyanins. Noticeably, bioactive compounds also contain glycosides, steroids, plant sterols, and peptides. The exploration, biosynthesis, and amplification of biological activities of natural and non-natural molecules have remained unchanged as one of the most exciting trends in biotechnological products.

Most of the bioactive compounds have been isolated from plants, microorganisms, marine organisms, and fungi. However, the yields of natural products are usually low and require time-consuming procedures for industrial production. Furthermore, chemical synthesis is a costly, harmful alternative and requires multi-step isolation and purification processes. Although plant molecular engineering has been significantly developed, using industrially preferred microorganisms is a promising approach for the biosynthesis of industrial products. In recent years, a wide variety of novel technologies for engineering plants and microbes have been developed to produce natural and non-natural compounds from renewable biomasses. Along with evolutionary engineering, metabolic engineering, and systems biology, synthetic biology is expected to further improve the productivity of the compounds. This topic focuses on providing an overview of the recent advances, emerging challenges, and future prospects of synthetic biology and metabolic engineering for the biomanufacturing of bioactive compounds.

High-value compounds have been synthesized and produced using various engineered platforms. For example, Liu et al. provided an overview of the microbial chassis for the production of fatty acid-derived chemicals (FACs). Interestingly, autotrophs (such as *Cupriavidus necator*, *Rhodococcus opacus*, *Synechococcus* sp. PCC 7002, and *Nostoc punctiforme*) can synthesize FACs from CO_2_ using chemical sources, light, and electric energy; while heterotrophs (as *Escherichia coli*, *Saccharomyces cerevisiae*, *Yarrowia lipolytica*, and *Aureobasidium*) were designed and engineered to produce FACs from organic carbon sources. Sajied et al. summarized the current *ex-planta* production of isoflavonoid *via* an artificial isoflavonoid biosynthesis pathway in engineered *E. coli* and *S. cerevisiae*. Furthermore, Wang et al. reviewed the fermentation processes in the production of water-soluble vitamins (vitamin B1, B2, B3, B5, B6, B7, B9, B12, and vitamin C) and fat-soluble vitamins (vitamin A/D/E and vitamin K) in microbial cell factories. Noticeably, Mutanda et al. summarized comprehensive information regarding the current biotechnological production of taxol in engineered plants (*A. thaliana*, *Nicotiana benthamiana*, *Nicotiana Sylvestris*), endophytic fungus (*Alternaria alternate*) as well as engineered microbes (*E. coli*, *S. cerevisiae*). They have provided a novel insight on the regulatory mechanism governing taxol biosynthesis by understanding the challenges with metabolic engineering and mining of transcriptomic data sets from *Taxus* species. The review demonstrated that plant genetic engineering has been significantly developed to improve the catalytic process and product specificity of the important compounds. Fu et al. demonstrated *Artemisia annua* L. with glandular trichomes can be used as a transgenic platform for high-level patchoulol production. Overexpressing farnesyl diphosphate synthase and patchoulol synthase genes along with alteration of the subcellular location resulted in increasing to 273 μg g^−1^ DW of patchoulol.

Overexpression of genes in biosynthetic pathway and inhibition of genes in competitive pathway plays a key role in engineered microorganisms for bioactive compound biosynthesis. Wang et al. engineered *Pseudomonas Chlororaphis* P3 to produce gentisate from 3-hydroxybenzoate (3-HBA) and 4-hydroxybenzoate (4-HBA). The chromosome-integrated synthetic pathway and blocking the key conversion steps resulted in the production of 365 mg L^−1^ of gentisate from 3-HBA. Similarly, Ma et al. reported the first report of the production of (+)-borneol using engineered *S. cerevisiae*. (+)-bornyl diphosphate synthase from *Cinnamomum burmanni* was modified by tailored truncation and adding Kozak sequences, then overexpressed in *S. cerevisiae* harboring reconstituted (+)-bornyl biosynthetic pathway. Moreover, Chen et al. reported the production of 2-keto-L-gulonic acid in an acetic acid bacterium *Gluconobacter oxydans*. They obtained 97 promoters and identified the activity of the strongest promoter (P_2703_) from genome of *G. oxydan*. After that, gene succinate dehydrogenase (*SDH*) was overexpressed under the control of P_2730_ in *G. oxydan* WSH-003. An engineered strain reached 3.7 g L^−1^ of 2-keto-L-gulonic acid. On another hand, activation of the silent biosynthetic gene clusters (BGCs) in the native host is an essential strategy to discover novel natural products. For example, Liu et al. reviewed the major approaches for activation of natural product BGCs in *Streptomyces*. They described the strategies to metabolic regulatory network for *in situ* activation of target BGCs, including promoter engineering, transcriptional regulation engineering, ribosome, and RNA polymerase engineering.

In general, the research topic collected excellent examples of advanced metabolic engineering assisted by synthetic biology. The engineered host in this research topic showed the ability to produce bioactive compounds on industrial fermentation. However, there is still need to develop advanced synthetic biology tools in both plant and microbial platforms. Despite information in databases, such as KEGG, BioCyc, MetaCyc, or BRENDA, and utilization of genomic editing tools as ZFNs, TALENs, or CRISPR/Cas system, having been rapidly increased in recent years, the efficient synthetic biology tools for non-conventional microbial platforms are still limited. These barriers have resulted in limitations of the titer, rate, and yield of bioactive compound products. In order to overcome the bottlenecks and further advances in engineered strains, Ramzi et al. provided an omics technology and machine learning (ML) platform as an efficient tool for optimizing biosynthetic pathways and enhancing the microbial production capacity. Moreover, ML-based synthetic biology-combined artificial intelligence (AI) has a promising approach to generate a super host with an enhanced metabolic pathway centered for industrial bioactive compound products. The intelligence-produced hosts are expected to not only generate novel biomolecules but also combine carbon-fixing autotrophs and heterotrophs with net-zero greenhouse gas emissions.

In summary, the current research topic summaries a valuable collection of articles focusing on recent development and application of synthetic biology and metabolic engineering for secondary metabolite production in plant and microbial platforms. Omics and ML-assisted synthetic biology and metabolic engineering are expected to play significant roles to overcome the emerging challenges faced in agricultural, medical, and environmental biotechnology.

